# Preparation of Highly Porous Thiophene-Containing DUT-68 Beads for Adsorption of CO_2_ and Iodine Vapor

**DOI:** 10.3390/polym13234075

**Published:** 2021-11-24

**Authors:** Songtao Xiao, Menglin Li, Haifeng Cong, Lingyu Wang, Xiang Li, Wen Zhang

**Affiliations:** 1 Department of Radiochemistry, China Institute of Atomic Energy, Beijing 102413, China; 79819cong@163.com (H.C.); 18810128827@163.com (L.W.); 18311361990@139.com (X.L.); 2 State Key Laboratory of Chemical Engineering, Tianjin Key Laboratory of Membrane Science & Desalination Technology, School of Chemical Engineering and Technology, Tianjin University, Tianjin 300350, China; 2020207432@tju.edu.cn

**Keywords:** DUT-68, alginate beads, CO_2_ capture, iodine adsorption

## Abstract

Zirconium-based metal-organic frameworks (Zr-MOFs) have great structural stability and offer great promise in the application of gas capture. However, the powder nature of MOF microcrystallines hinders their further industrial-scale applications in fluid-phase separations. Here, Zr-based DUT-68 was structured into nontoxic and eco-friendly alginate beads, and the gas capture properties were evaluated by CO_2_ and volatile iodine. DUT-68 beads were synthesized via a facile and versatile cross-linked polymerization of sodium alginate with calcium ions. The composite beads keep the structural integrity and most of the pore accessibility of DUT-68. The resulting DUT-68@Alginate (2:1) porous bead processes a surface area of 541 m^2^/g and compressive strength as high as 1.2 MPa, and the DUT-68 crystals were well-dispersed in the alginate networks without agglomeration. The DUT-68@Alginate bead with a 60% weight ratio of MOFs exhibits a high carbon dioxide capacity (1.25 mmol/g at 273 K), as well as an excellent high adsorption capacity for iodine, reaching up to 0.65 g/g at 353 K. This work provides a method to construct thiophene-contained composite beads with millimeter sizes for the capture of gases in potential industrial applications.

## 1. Introduction

The fossil oil is one of the most crucial energy sources to develop the society and sustain our lives. The large-scale use of fossil oil could generate excessive greenhouse gases, in which CO_2_ is the main contribution and should be removed. At the same time, the development of carbon-free nuclear energy is regarded as a potential strategy to produce clean energy with the advantages of high energy density, low operation cost and little CO_2_ emission. However, in the nuclear industry, the spent fuel rods unloaded from the reactors need to be treated in the fuel reprocessing plants, in which complicated exhaust gas mixtures including radioactive iodine vapor could be discharged. The above iodine vapor should be removed completely and disposed securely to ensure the sustainable usage of nuclear energy.

Metal-organic frameworks (MOFs), composed of metal nodes and organic linkers, have emerged as new inorganic-organic hybrid materials and exhibited high surface areas, structure diversity, tailorable pore sizes, and tailorable functionality. Recently, MOFs have attracted more and more interest and potential in the applications of gas storage and separation, fluorescence detection, heterogeneous catalysis, ion conductivity, and so on. Particularly, MOFs with Zr metal nodes (Zr-based MOFs) are regarded as promising materials for industrial locations because of their particular robustness. Bearing the high connectivity and strong hard Lewis acidity, Zr clusters could be regarded as a versatile node to coordinate multiple organic carboxylate linkers with various topologies. In fact, several Zr-based MOFs have been used for the capture of CO_2_ and iodine vapor, such as UiO-66 [1], MOF-808 [2], NU-1000 [3]. However, these MOFs with aromatic-ring linkers usually have low adsorption capacity for CO_2_ due to the weak affinity because these aromatic rings and CO_2_. Introducing of heteroatoms, such as nitrogen, fluorine and sulfur in the rings, could enhance the polarity and basicity of frameworks, which is beneficial to increase the Lewis acid-base interactions between rings and CO_2_ [4,5,6]. Hence, to enhance their adsorption capacity, several functional groups have been introduced into Zr-MOFs, such as amino groups, fluorinated alkyl groups. However, the integration of these groups by post-modification methods could reduce the pore volumes and change their structure stability when interacting with guest species. Using the functionalized linkers to synthesize MOFs directly is another way to incorporate functional groups into MOFs without sacrificing pore volumes. Several functionalized linkers have been used to synthesize MOFs for CO_2_ adsorption [7], such as triazolyl isophthalate linkers [8], imidazolate links [9], and so on.

Thiophene groups are sulfur-containing ligands and could be used as the linkers to construct the stable Zr-MOFs, such as DUT-67, DUT-68 and DUT-69 [10,11]. Besides, several polymers with thiophene ligands have been reported for the uptake of CO_2_ [4,12], and DUT-68 has also exhibited an excellent iodine uptake performance due to the electron transfer from thiophene ligands to iodine molecules [13]. However, there is little study about the uptake of CO_2_ using Zr-MOFs with thiophene ligands. Furthermore, for practical industrial adsorption of CO_2_ and iodine vapor, structuring fine MOF powders into beads is necessary to break through the operation limitations, such as pressure drop, inhomogeneous flow, frangibility, agglomeration, clogging, leakage and mass loss. Therefore, the fabrication of resistant beads from MOF powders remains of large interest to the field of gas capture, such as for CO_2_ and iodine uptake applications.

The aim of this study is to combine the adsorption advantages of Zr-based MOFs with thiophene ligands and the adaptability of polymers to create robust beads, which are used to capture CO_2_ and iodine vapor. The stable DUT-68 was selected due to its cage-typed mesopores could help to keep gas molecules kinetically trapped due to the confinement effect [13,14,15]. The alginate polymer matrices were selected to prepare the millimeter-sized composite beads using an easy and scalable precipitation method [16]. Different amounts of DUT-68 were incorporated into the composite beads, and the morphology, composition and structure of these DUT-68@Alginate beads were studied systematically. The CO_2_ and iodine adsorption uptake performance were determined. The role of alginate chains for the adsorption properties of CO_2_ and iodine was also discussed. This work highlights the millimeter-sized alginate beads with highly dispersed thiophene-containing MOFs for the capture of CO_2_ and iodine gases.

## 2. Experiments

### 2.1. Synthesis of DUT-68@Alginate Beads

Sodium alginate (low viscosity) and anhydrous calcium chloride (AR) were purchased from Heowns Science and Technology Ltd., Tianjin, China. All reagents were used as received without further purification. DUT-68 was synthesized hydro-thermally according to the reported methods [11]. Before use, the DUT-68 powder was dried at 393 K under vacuum for 12 h. The production of spherical beads is as follows. The DUT-68 was dispersed in 5 mL of 20 mg/mL sodium alginate aqueous solution, and then the obtained MOF/alginate slurry was stirred at room temperature for 1 h. Meanwhile, the curing solution was prepared by dissolving 2% (*w/v*) of anhydrous calcium chloride in 50 mL deionized water. Then, the alginate suspension was added into the curing solution drop by drop using a syringe pump. After shaping, the beads were collected and washed with distilled water several times. Then, the obtained DUT-68@Alginate beads were dried using a freezing dryer. The samples with DUT-68/sodium alginate weight ratios of 1:1 and 2:1, are labeled as DUT-68@Alginate (1:1) and DUT-68@Alginate (2:1), respectively.

### 2.2. Characterization

The N_2_ adsorption-desorption isotherms of DUT-68@Alginate beads were recorded using a surface analyzer at 77 K (Micromeritics ASAP2460, Micromeritics Instrument Corporation, Norcross, GA, USA). Before the measurement, the beads were degassed at 393 K under vacuum for 12 h. The CO_2_ adsorption-desorption experiments were also performed on the Micromeritics ASAP2460 instrument at 273 K or 298 K. The field emission scanning electron microscopy (SEM) images of beads were obtained with a Regulus 8100 scanning microscope with Energy Dispersive Spectroscopy (EDS). Powder X-ray diffraction (PXRD) patterns of the beads were taken on a Bruker/ D8-Focus Cu Kα diffractometer with a scan rate of 7.5 °/min. The thermogravimetric curves of the beads were recorded using the thermal gravimetric analyzer (TG209F1 Libra, NETZSCH Group, Selb, Germany) with a heating rate of 10 °C/min under the N_2_ atmosphere. The elemental analysis of sulfur was conducted using an elemental analyzer (Elementar vario el III, Langenselbold, Germany). The compressive strength of the beads was measured using a Compression strength tester, the initial diameter of beads is 3 mm. The final thickness of the compressed beads is set to 1 mm, and the applied force at this point was used to calculate the compressive strength. The X-ray photoelectron spectroscopy (XPS, Thermo Scientific Escalab 250Xi, Waltham, MA, USA) of the beads was also measured to analyze the elemental chemical bonding states. The procedure of iodine adsorption by DUT-68@Alginate beads was carried out according to the previous work [13,17]. Briefly, sealed bottles with 20 mg beads in a glass vial and 200 mg solid iodine were kept at 358 K in the oven and weighted at different times. The iodine adsorption capacity was calculated using the weighing method.

## 3. Results and Discussion

**Figure 1:**
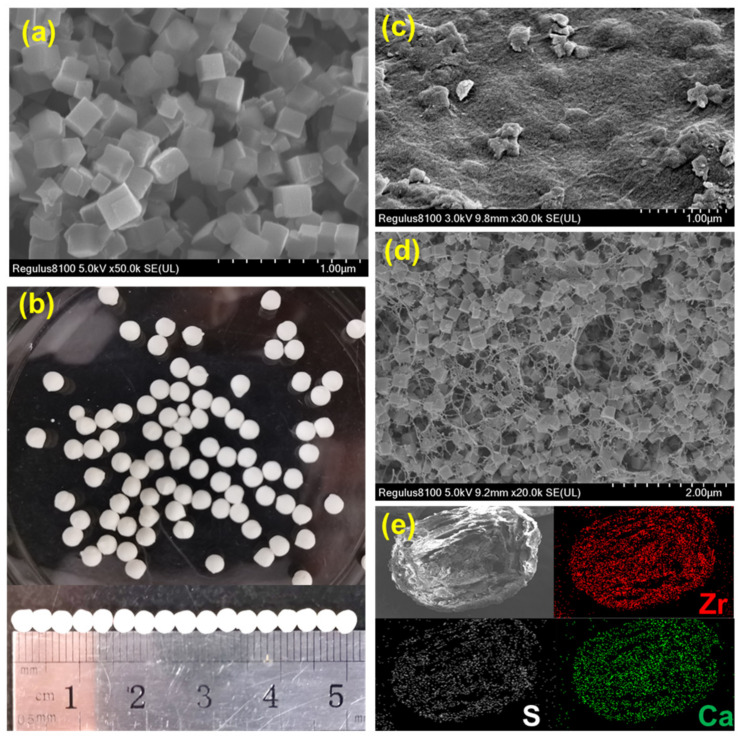
(**a**) SEM image of DUT-68; (**b**) Optical image of DUT-68@Alginate; (**c**) SEM image of the outside surface of DUT-68@Alginate; (**d**) Cross-section SEM image of DUT-68@Alginate; (**e**) EDS mapping image of DUT-68@Alginate.

The SEM image of DUT-68 exhibits its regular cubic morphology with a size of about 2 um (Figure 1a). The DUT-68@Alginate beads were prepared based on crosslinking of alginate-Ca^2+^ at the room temperature. The DUT-68 powder was integrated with alginate polymers by adding the mixture of MOFs and sodium alginate solution into the CaCl_2_ solution dropwise. The obtained DUT-68@Alginate beads have a uniform diameter of 3 mm (Figure 1b). In Figure 1c, there are some MOFs scattered on the undulating outside-surface of the beads. In the cross-section SEM image of DUT-68@Alginate, the cubic DUT-68 crystallites could be clearly observed inside the porous beads (Figure 1d), suggesting the morphology of DUT-68 is kept during the preparation process. Besides, EDS mapping signals from Ca and Zr were observed throughout the bead. This implies that the DUT-68 has a homogeneous distribution throughout the beads (Figure 1e). The compressive strength of DUT-68@Alginate beads was also measured. The compressive strengths of the DUT-68@Alginate (2:1), DUT-68@Alginate (1:1) and control alginate beads are 1.2 MPa, 0.9 MPa and 0.7 MPa, respectively. The enhanced compressive strength of composite beads is beneficial for the filling of the fixed bed.

**Figure 2:**
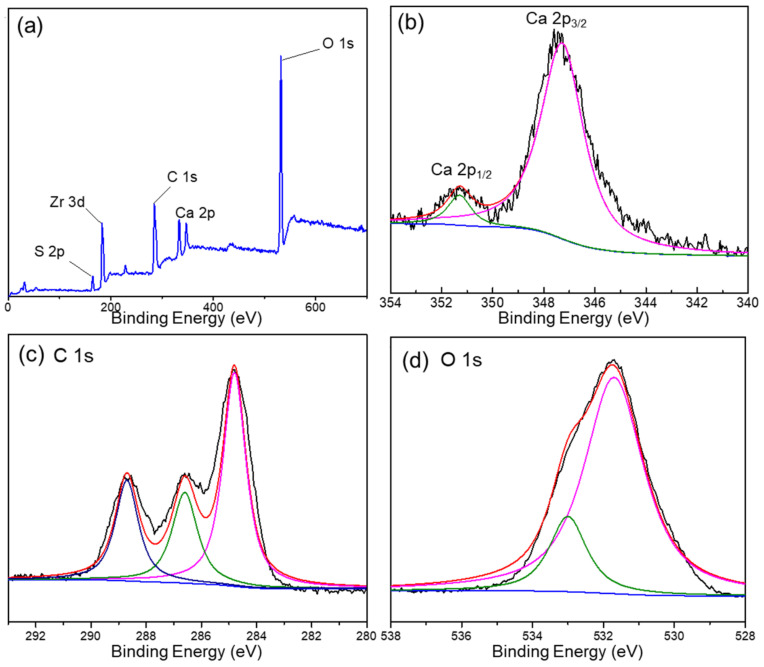
(**a**) XPS of DUT-68@Alginate (2:1) and the high-resolution XPS spectra of (**b**) Ca, (**c**) C and (**d**) O elements.

The XPS spectra of the DUT-68@Alginate (2:1) bead were recorded to confirm the polymerization reaction and integration of MOFs. In Figure 2a, both Zr and S elements are presented with a Zr/S atomic ratio of about 1.3, suggesting the incorporation of integrated DUT-68 into the composite beads. The weight percent of DUT-68 in DUT-68@Alginate (2:1) beads, calculated with the S element contents from the elemental analysis (shown in Table 1), are very close to the initial feed ratios of DUT-68 and alginate salts. The peak of Ca 2p3/2 is located at 347.3 eV, close to the BE value found in CaCO_3_, which could be attributed to the bonding of O-Ca-O between Ca^2+^ and carboxylate ligands [18]. The C 1s spectrum of the composite exhibits three peaks at 284.7 eV, 286.6 eV, and 288.7 eV, which corresponds to the sp^3^-C, C=O, and O-C=O bond, respectively. The O 1s spectrum can be assigned to two peaks located at around 531.7 eV and 533 eV, which represent C=O bond and C-OH, respectively. The above high-resolution XPS spectra indicate the successful cross-linking between Ca^2+^ and the carboxyl groups of alginate salts.

**Figure 3:**
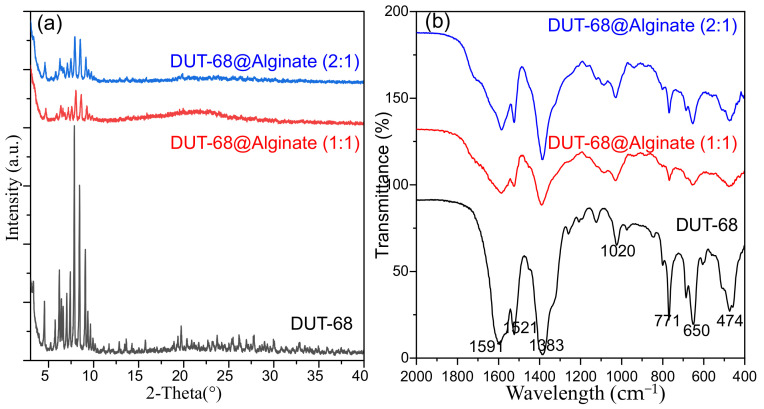
(**a**) PXRD patterns and (**b**) FTIR of DUT-68 and DUT-68@Alginate beads.

The structural integrity of DUT-68 after being encapsulated into beads was confirmed by PXRD patterns. From Figure 3a, the peaks of DUT-68 are kept in the composite beads, with a decrease in relative intensities, due to the dispersive distribution of DUT-68 in the beads. The FTIR spectra were collected to illustrate the functional groups in the composite beads (Figure 3b). the peaks at 1383 cm^−1^ and 1591 cm^−1^ could be assigned to the symmetric and asymmetric stretching vibration of the coordinated carboxylate groups on TBC linkers [19]. The peaks at 650 cm^−1^ and 771 cm^−1^ could be attributed to the vibrations of thiophene rings [20]. The FTIR spectra suggest the DUT-68 keeps its original functional group in the composite beads.

**Figure 4:**
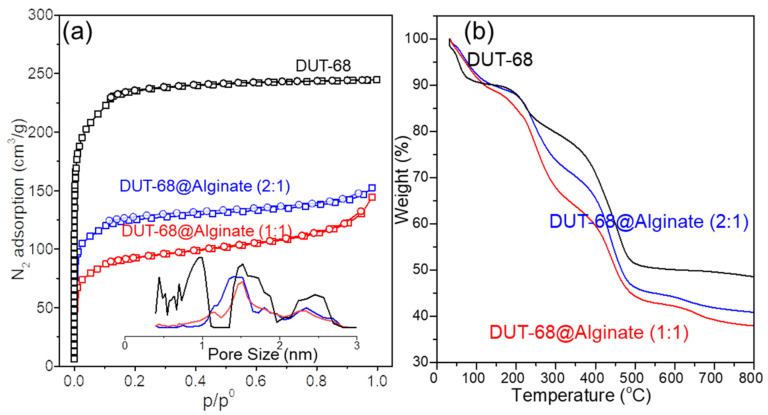
(**a**) N_2_ adsorption-desorption isotherms with the NLDFT pore-size distribution and (**b**) thermogravimetric curves of DUT-68 and DUT-68@Alginate beads.

The pore accessibility was tested by a nitrogen adsorption-desorption experiment, showing a typical type I isotherm with a microporous structure in DUT-68 and two beads (Figure 4a). The BET surface areas of DUT-68 powder, DUT-68@Alginate (2:1), and DUT-68@Alginate (1:1) are 917 m^2^/g, 541 m^2^/g and 407 m^2^/g, respectively. However, the alginate polymer exhibits an adverse effect on the porosity of the MOFs. After being embedded in the alginate matrix, the expected BET surface areas of DUT-68 become lower by about 5–8%, when considering the mass of MOFs, implying a decrease in the accessible area of N_2_ molecules. That could be attributed to the interaction between alginate and Zr-nodes, which could form a thin layer covering the large pores on the surface of MOFs [21]. The pore volumes of DUT-68 powder, DUT-68@Alginate (2:1), and DUT-68@Alginate (1:1) are 0.65 cm^3^/g, 0.31 cm^3^/g and 0.28 cm^3^/g, respectively, calculated by the non-local density functional theory (NLDFT) approach. Hence, most of the high specific surface areas and pore volumes of DUT-68 remain after being embedded in the alginate matrix.

The thermogravimetric curves (Figure 4b) of the DUT-68 exhibit a remarkable weight loss before 100 °C, which could be attributed to the departure of physically adsorbed water. Between 200 °C and 300 °C, the DUT-68 undergoes the departure of DMF, structural water and anchored carboxylate ligands [11,22]. At about 400 °C, the decomposition of TDC linkers takes place, and there is a sharp weight loss at this stage. After 500 °C, the weight loss is negligible and ZrO_2_ remains in the residue. The thermogravimetric curves of DUT-68@Alginate beads have a similar sharpness to that of DUT-68, and lower weight residue weights at 800 °C. After 200 °C, the pyrolyzation takes place in the polysaccharide chains of alginates, remaining carbon residues and CaCO_3_ at last. The thermogravimetric curves suggest that the operating temperature of DUT-68@Alginate beads should be less than 200 °C to avoid their decomposition.

**Figure 5:**
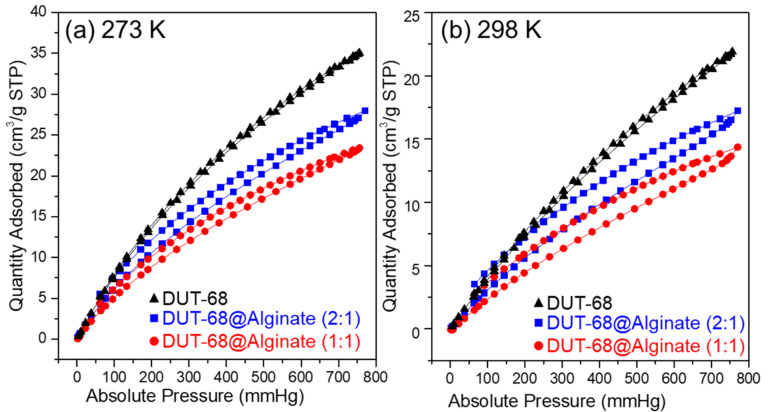
CO_2_ adsorption-desorption curves of DUT-68 and DUT-68@Alginate beads at (**a**) 273 K and (**b**) 298 K.

As we know, the frameworks with polar groups could enhance their CO_2_ binding energy, resulting in a high affinity toward CO_2_. In DUT-68, the thiophene-based heterocycle linkers could interact with CO_2_ strongly due to the sulfur atoms improving the polarity and basicity of frameworks. Besides, the carboxylate-linkers of Zr-based MOFs are usually rich in hydrogen bonds and dipole-quadrupole interactions between CO_2_ and the groups of porous frameworks [23]. We performed CO_2_ uptake measurements at 298 K and 273 K to examine their CO_2_ uptake abilities, as presented in Figure 5. The results revealed that DUT-68 displayed a CO_2_ adsorption capacity of 1.56 mmol/g at 273 K and 0.98 mmol/g at 298 K. After being incorporated into alginate beads, DUT-68 remains their CO_2_ uptake performance, and the resulted DUT-68@Alginate (2:1) and DUT-68@Alginate (1:1) show the CO_2_ adsorption capacity of 1.25 mmol/g and 1.05 mmol/g at 273 K, respectively. The CO_2_ adsorption capacity of DUT-68 and DUT-68@Alginate beads, compared with other porous materials with thiophene ligands, are listed in Table 1. We could see that the thiophene-based MOFs, DUT-68, Zn_2_(tdc)_2_DABCO and Cu_6_(DDC)_3_, exhibit higher CO_2_/S molar ratios than other amorphous thiophene-based porous polymers, suggesting their more efficient usage of thiophene ligands. Besides, DUT-68@Alginates have a larger CO_2_/S molar ratio than DUT-68, suggesting that the alginate matrix may also play a role in CO_2_ adsorption. As we know, Zr metal nodes could interact with the alginate chains and thus form a thin layer covering the large pores on the surface of MOFs. Hence, the alginate layer with abundant carboxyl and hydroxyl groups could facilitate the transport of CO_2_ into the inside of MOF nano-particles, and also interact with CO_2_ molecules synergistically to enhance its capture capacity [24,25,26,27].

**Figure 6:**
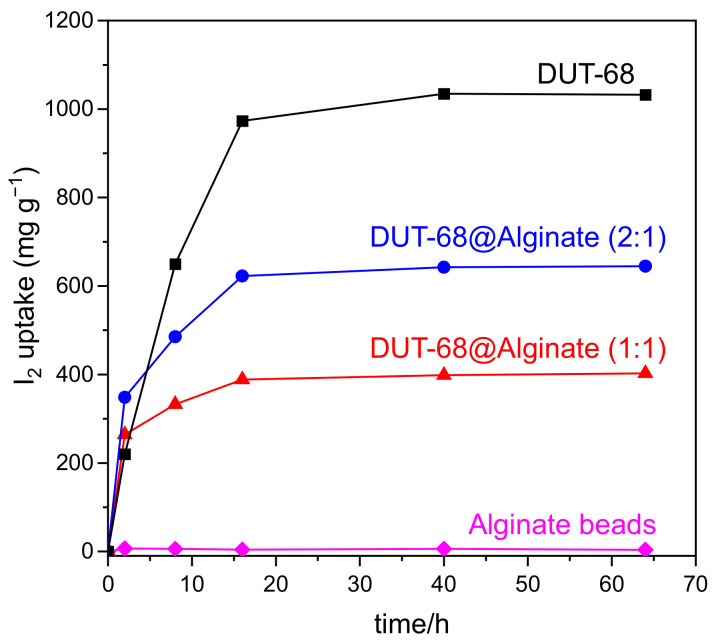
Iodine uptake curves of DUT-68 and DUT-68@Alginate beads at 353 K.

The iodine adsorption curves of the DUT-68@Alginate beads were measured in saturated iodine vapor at 353 K. As shown in Figure 6, the pure alginate bead shows little iodine uptake capacity, while DUT-68 displays a high iodine uptake capacity of 1.03 g/g at equilibrium. DUT-68@Alginate (2:1) shows an uptake capacity of 0.65 g/g at equilibrium, about 63% of that for DUT-68, suggesting that the iodine uptake capacity of DUT-68 powder was remained about 95% after embedding in the alginate beads. For DUT-68@Alginate (1:1), it shows an iodine uptake capacity of about 41% of that for DUT-68, suggesting that the iodine uptake of DUT-68 remained about 82% after embedding in the beads. These results suggest that the DUT-68@Alginate (2:1) bead has a higher utilization of DUT-68 than the DUT-68@Alginate (1:1) bead. Different from the CO_2_ molecules, the iodine molecules with much larger sizes need larger pore sizes to diffuse into the MOFs. However, the increased alginate molecular chains may coat the surface of MOFs and thus block parts of the porous channels of DUT-68, resulting in a reduced utilization of MOF materials. Besides, it is worth noting that in the first two hours, the iodine adsorption capacity of DUT-68 is lower than the DUT-68@Alginate beads, suggesting the high-dispersed MOF microcrystals supported by the alginate matrix has a better contact with the iodine vapor and could reduce the diffusion time.

## 4. Conclusions

In this work, porous DUT-68@Alginate beads have been fabricated using an easy polymerization reaction between alginate and calcium ions. The uniform size of the beads is about 3 mm, and the DUT-68 is well-dispersed in the alginate networks without agglomeration. The alginate matrix also provides rigid support for DUT-68 with high pressure strength (1.2 MPa). After embedding in alginate beads, the surface area and pore volume of DUT-68 decrease by 5–8%. However, the CO_2_ adsorption capacity is enhanced due to the synergistic effect of alginate polymers. The composite bead exhibits a CO_2_ adsorption capacity of 1.25 mmol/g at 273 K, with a highly efficient usage of thiophene ligands in the MOFs. Meanwhile, the composite bead also shows an iodine uptake capacity of 0.65 g/g at 353 K, and the alginate support is beneficial to the quick contact between DUT-68 and iodine vapor.

## Figures and Tables

**Table 1:** Performance comparison of CO_2_ adsorption using adsorbents with thiophene ligands.

Adsorbents	S_BET_ (m^2^/g)	V_total_ (cc/g)	Temp. (K)	Pressure (bar)	S Element Content (wt.%)	CO_2_ Adsorption Capacity (mmol/g)	CO_2_/S Molar Ratio
HMC-1 [12]	855	0.297	273	1	27.1	5.00	0.59
SCMP-COOH@1 [10]	911		273	1	16.86	2.14	0.41
CTF-DCBT [4]	500	0.26	298	1	12.61	1.69	0.43
CK-COP-2 [28]	615	0.68	273	1	24.16	2.13	0.28
Zn_2_(tdc)_2_DABCO [29]	1553	0.68	273	1	10.9	6.82	2.17
Cu_6_(DDC)_3_ [30]	2410	0.98	273	1	5.13	8.04	5.02
DUT-68	917	0.65	273	1	6.58	1.56	0.76
DUT-68@Alginate (2:1)	541	0.31	273	1	4.32	1.25	0.93
DUT-68@Alginate (1:1)	407	0.28	273	1	3.32	1.05	1.01
